# Evaluation of Machine Learning Approaches to Predict Soil Organic Matter and pH Using vis-NIR Spectra

**DOI:** 10.3390/s19020263

**Published:** 2019-01-11

**Authors:** Meihua Yang, Dongyun Xu, Songchao Chen, Hongyi Li, Zhou Shi

**Affiliations:** 1 Institute of Agricultural Remote Sensing and Information Technology Application, College of Environmental and Resource Sciences, Zhejiang University, Hangzhou 310058, China; 0015862@zju.edu.cn (M.Y.); xudongyun@zju.edu.cn (D.X.); 2 Department of Environmental Engineering, Yuzhang Normal University, Nanchang 330103, China; 3 The Institute National de la Recherche Agronomique (INRA), Unité InfoSol, 45075 Orléans, France; songchao.chen@inra.fr; 4 Department of Land Resource Management, Jiangxi University of Finance and Economics, Nanchang 330013, China; lihongyi1981@zju.edu.cn; 5 Key Laboratory of Spectroscopy Sensing, Ministry of Agriculture, Hangzhou 310058, China

**Keywords:** machine learning approaches, vis-NIR spectra, paddy soil, soil organic matter, pH

## Abstract

Soil organic matter (SOM) and pH are essential soil fertility indictors of paddy soil in the middle-lower Yangtze Plain. Rapid, non-destructive and accurate determination of SOM and pH is vital to preventing soil degradation caused by inappropriate land management practices. Visible-near infrared (vis-NIR) spectroscopy with multivariate calibration can be used to effectively estimate soil properties. In this study, 523 soil samples were collected from paddy fields in the Yangtze Plain, China. Four machine learning approaches—partial least squares regression (PLSR), least squares-support vector machines (LS-SVM), extreme learning machines (ELM) and the Cubist regression model (Cubist)—were used to compare the prediction accuracy based on vis-NIR full bands and bands reduced using the genetic algorithm (GA). The coefficient of determination (R^2^), root mean square error (RMSE), and ratio of performance to inter-quartile distance (RPIQ) were used to assess the prediction accuracy. The ELM with GA reduced bands was the best model for SOM (SOM: R^2^ = 0.81, RMSE = 5.17, RPIQ = 2.87) and pH (R^2^ = 0.76, RMSE = 0.43, RPIQ = 2.15). The performance of the LS-SVM for pH prediction did not differ significantly between the model with GA (R^2^ = 0.75, RMSE = 0.44, RPIQ = 2.08) and without GA (R^2^ = 0.74, RMSE = 0.45, RPIQ = 2.07). Although a slight increase was observed when ELM were used for prediction of SOM and pH using reduced bands (SOM: R^2^ = 0.81, RMSE = 5.17, RPIQ = 2.87; pH: R^2^ = 0.76, RMSE = 0.43, RPIQ = 2.15) compared with full bands (R^2^ = 0.81, RMSE = 5.18, RPIQ = 2.83; pH: R^2^ = 0.76, RMSE = 0.45, RPIQ = 2.07), the number of wavelengths was greatly reduced (SOM: 201 to 44; pH: 201 to 32). Thus, the ELM coupled with reduced bands by GA is recommended for prediction of properties of paddy soil (SOM and pH) in the middle-lower Yangtze Plain.

## 1. Introduction

As a major soil type, paddy soils are widely distributed in China, with an area of about 30 million hm^2^. This accounts for 29% of the cultivated land in China, especially in the Yangtze River Delta and southern China [1]. Therefore, it is of great importance to evaluate and monitor the quality of paddy soils. In southern China, soil organic matter (SOM) and pH are important components in soil quality assessment as the former is directly related to crop yield and the latter to food security. Therefore, rapid, accurate and non-destructive assessment of SOM and pH is vital to soil fertility evaluation and monitoring under conventional cropping systems in large areas [1]. However, conventional laboratory measurement of soil properties is time-consuming, tedious and cannot be applied to large areas.

Visible near-infrared spectroscopy (vis-NIR) has become increasingly popular as an alternative to conventional laboratory analyses because it is rapid, non-destructive, cost-effective, does not require hazardous chemicals, and enables several soil properties to be simultaneously estimated from a single spectrum [2]. When vis-NIR radiation interacts with a soil sample, we can detect the overtones and combinations of fundamental molecular vibrations, such as O–H, C–H, N–H and C=O groups [3]. Vis-NIR has been used to predict soil chemical and physical properties, particularly for SOM, texture, and clay mineralogy [4].

However, soil vis-NIR spectra are largely nonspecific because of the overlapping absorption of soil constituents. Complex absorption patterns generated from soil constituents and quartz need to be mathematically extracted from the spectra [4]. Partial least squares regression (PLSR) is a commonly used linear model; however, there are many nonlinear relationships between spectral data and target soil characteristics in nature [5,6]. Therefore, some non-linear machine learning techniques, including artificial neural networks (ANN), support vector machine regression (SVMR), least square-support vector machines (LS-SVM), random forest and the Cubist regression model (Cubist) have been used [7,8,9,10,11,12,13]. Moreover, soil is a complex mixture that consists of water, air, and organic and inorganic mineral matter of variable origins, so it is difficult to achieve universal acceptance with the same calibration techniques. Some researchers [9,10,14] have shown that machine learning techniques lead to satisfactory results for the prediction of soil organic carbon (SOC) and pH in a large range area. Extreme learning machines (ELMs), the emergent machine learning technique put forward by Huang et al. [15], have been used extensively over the past several years because of their good generalization performance and extremely fast learning speed. In addition, the large number of spectral variables in most data sets need to eliminate unrelated variables to provide insight into the important wavelengths related to soil properties and enable their use in prediction of soil properties.

Therefore, this study was conducted to investigate paddy fields in the Yangtze Plain, China. The specific goals of this study were to: (i) explore the important wavelengths of vis-NIR in SOM and pH predictions; (ii) compare the performance of linear (PLSR) and non-linear (least square-support vector machines, LS-SVM; extreme learning machines, ELM; Cubist regression model, Cubist) models for predicting SOM and pH.

## 2. Materials and Methods

### 2.1. Study Area and Soil Sampling

**Figure 1:**
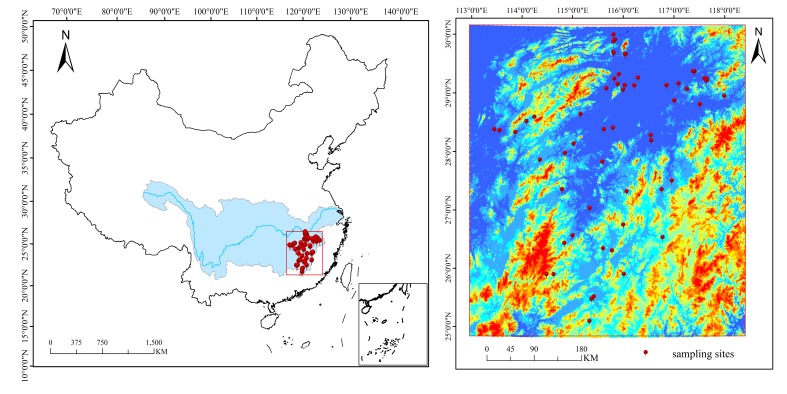
Location of sampling sites.

The study was conducted in the middle-lower Yangtze Plain, which includes Jiangsu, Zhejiang, Jiangxi and Hunan. The parent material of the soils is alluvial deposits from the Yangtze River and its tributaries and the main soil type of the study area is paddy soil, which is a kind of Anthrosol in Chinese Soil Taxonomy. We selected 57 paddy fields with an area of more than 0.6 km^2^, and 8–10 sampling sites for each field were selected, with a total of 523 soil samples ultimately being collected (Figure 1). The soil type of samples and the parent material can be seen in Table 1. These soil samples were air-dried, ground and sieved to less than 2 mm. Stones and plant residues were removed. Each sample was divided by the quartering method into two portions, one for laboratory chemical analysis and the other one for spectral measurements. The SOM content was measured by the potassium dichromate volumetric method, soil pH was determined in a slurry of soil and water at a ratio of 1:2.5 using an electronic pH meter.

### 2.2. Spectroscopic Measurement and Pre-Processing of Spectra

The vis-NIR spectra were measured with an ASD FieldSpec^®^ Pro FR spectrometer (Analytical Spectral Devices Inc., Boulder, CO, USA) using a high-intensity contact probe with a spectral range of 350 to 2500 nm and a spectral resolution of 1 nm. Each sample was placed in a petri dish (10 cm diameter and 1.5 cm depth), after which the spectrometer was calibrated using a Spectralon^®^ panel with 99% reflectance. We measured three replicates of spectra for each sample and each replicate consisted of 10 internal scans. The resulting 30 spectra were averaged into one to represent each sample.

A spectral range of 400 to 2400 nm was used because the spectra outside this range had a lot of noise. The reflectance spectra (R) were transformed to absorbance (A = log10(1/R)), then resampled to 10 nm to reduce dimensionality. The spectra of 523 samples were split into calibration (350 samples) and validation (173 samples) sets using the Kennard–Stone algorithm [16]. We used the continuum-removed spectrum [17] of the mean of all samples to help interpret the main absorption features in the spectra.

We used the genetic algorithm (GA) to select an optimal subset of spectral bands in the calibration process. Many studies [18,19,20] have demonstrated the importance of GA wavelength selection in the calibration step to avoid the selection of random correlation and irrelevant variables. Wavelength selection using GA can improve the robustness of multivariate calibrations without loss of prediction capacity, and furthermore, provides useful information about the chemical system [11]. We used the method of genetic algorithm (GA) and partial least squares regression (GA–PLS) for feature selection. In this study, the parameter values of the GA were set as follows based on Leardi et al. [21] and Shi et al. [22]: max generation of 100, population size of 64, mutation rate of 0.01 and replicate run of 10. The fitness function was determined according to the root mean square error of cross-validation (RMSECV) of the partial least squares. For this we used the PLS_Toolbox 8.5.1 (Eigenvector Research Inc., Wenatchee, WA, USA) in MatLab (R2016A, The MathWorks Inc., Natick, MA, USA).

### 2.3. Multivariate Regression Models

#### 2.3.1. Partial Least Squares Regression (PLSR)

Partial least squares regression (PLSR) is a linear regression model widely used in the quantitative analysis of diffuse reflectance spectra in soil [23]. This method uses a latent variable approach to model covariance structures in two projected spaces of the predicted and observed variables [24]. The optimum number of latent variables would be the number that yields the minimum prediction error sum of squares using cross-validation of the calibration set. For PLSR we used the R package ‘pls’ [25] of R 3.3.3 [26].

#### 2.3.2. Least Squares-Support Vector Machines (LS-SVM)

The LS-SVM method employs classification and regression analysis to solve linear and nonlinear multivariate problems [27]. The LS-SVM method uses linear equations instead of convex quadratic programming for classical SVMs. Additionally, the LS-SVM uses a Kernel function of Gaussian radial basis function (RBF). We used a systematic grid search method to optimize the parameters C and γ of the RBF. The optimal model parameters were determined by the lowest RMSE in the calibration set by leave-one-out cross-validation. This was done with MatLab.

#### 2.3.3. Cubist Regression Model

The Cubist model is a data mining technique and an extension of the M5 model tree developed by Quinlan [28]. Cubist is a rule-based regression where a model tree is first created and then reduced to a series of rules. These rules partition samples according to their spectra, and a unique linear model is then applied to predict the target variable. In addition, Cubist can utilise boosting (committees) and adjust its predictions using neighbours from within the training data set (neigbours). More details on Cubist and its implementation can be found in Viscarra Rossel and Webster and Minasny and McBratney [9,29]. The committees and neighbours were determined by the lowest RMSE in the calibration set by leave-one-out cross-validation. For Cubist we used the R package ‘Cubist’ [30] of R 3.3.3 [26].

#### 2.3.4. Extreme Learning Machine

**Figure 2:**
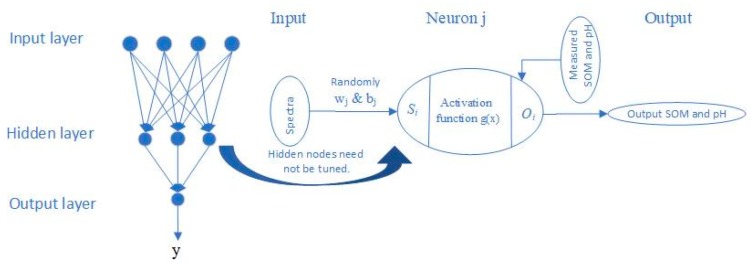
Structure of ELM (*w*_j_, weighting; *b*_j_, biases).

The extreme learning machine (ELM) is a generalized single-hidden layer feedforward network (SLFN) with a weight and hidden layer threshold in the first layer that are randomly assigned and a weight in the output layer that is calculated directly by the least-squares method. The entire learning process is completed in one round, with no iterations required; therefore, this algorithm performs at extremely fast learning speed. The simplified scheme can be found in Figure 2.

Algorithm ELM: For N distinct samples (***x_i_***, ***t_i_***), where *x_i_* = ${\left[x_{i1},x_{i2},\ldots ,x_{in}\right]}^{T}$ ∈ **R**^n^, ***x_i_*** were soil spectra, while ***t_i_*** were the observed values of either SOM or pH.

Given a hidden node number $\hat{N}$, the activation function is defined as follows:

(1) $$g(x)=\sum_{j=1}^{\hat{N}}\beta_{j}g_{j}(x_{i})=\sum_{j=1}^{\hat{N}}\beta_{j}g(w_{j}\cdot x_{i}+b_{j})=o_{i},\;i=1.2,\ldots ,N;\;j=1,2,\ldots \hat{N}$$

where ***w_j_*** ∈ **R**^n^ is the weight vector connecting the input nodes to the *j*th hidden node and ***b_j_*** ∈ **R** is the threshold of the *j*th hidden node, *β_j_* ∈ **R** represents the weight vector connecting the *j*th hidden node and the output nodes. To approach the real results of the training data infinitely, the prediction result *o_i_* must be consistent with the real result ***t_i_***, in which case $\sum_{i=1}^{\hat{N}}\|o_{i}-t_{i}\|=0$. Under these conditions, Equation (1) can be expressed as follows: $\sum_{i=1}^{N}\beta_{j}g(w_{j}\cdot x_{i}+b_{j})=t_{i}$ which is represented by a matrix:

**Hβ** = **T** (2)

where:

(3) $$H={\left[\begin{array}{ccc} g\left(w_{1}\cdot x_{1}+b_{1}\right) & \cdots & g\left(w_{\hat{N}}\cdot x_{1}+b_{\hat{N}}\right)\\ \vdots & \ldots & \vdots \\ g\left(w_{1}\cdot x_{N}+b_{1}\right) & \cdots & g\left(w_{\hat{N}}\cdot x_{N}+b_{\hat{N}}\right) \end{array}\right]}_{N\times \hat{N}},\beta ={\left[\begin{array}{c} \beta_{1}\\ \vdots \\ \beta_{\hat{N}} \end{array}\right]}_{\hat{N}\times 1}\mathrm{and}\;T={\left[\begin{array}{c} t_{1}\\ \vdots \\ t_{N} \end{array}\right]}^{T}$$

when input weight *w_j_* ∈ **R**^n^ and bias *b_j_* ∈ **R** are randomly assigned, the output matrix **H** in the hidden layer can be calculated by ELM, after which the output weight **β** is calculated by $\hat{\beta }=H^{†}T$ where $H^{†}$ is the Mosse-Penrose generalized inverse of **H**.

The ELM regression ability varies significantly with the number of initial hidden neurons. To select the number of hidden neurons, we conducted an experiment by varying the number of hidden neurons from 1 to 120 in steps of 1. The optimum number of initial hidden neurons would be the number that produced the minimum prediction error sum of squares for cross-validation of the calibration set. This was done in MatLab.

### 2.4. Model Evaluation

We compared the measured values vs. the predicted values of the cross-validation in the calibration and validation data sets using simple linear regression. The predictability of the different calibration models was evaluated by the coefficient of determination (R^2^), mean error (ME), root mean square error (RMSE) and ratio of performance to inter-quartile distance (RPIQ), which were suggested for assessing vis-NIR model performance by Bellon-Maurel et al. [31]. Generally, larger values of R^2^ and RPIQ and smaller RMSE indicate better model performance.

## 3. Results

### 3.1. Descriptive Statistics of the Soil Properties

Table 2 shows a summary of statistics describing the SOM and pH for the total, calibration, and validation datasets. The SOM ranged from 2.44 to 60.50 g kg^−1^ and the pH ranged from 3.92 to 8.60 for the whole dataset. The coefficient of variation (CV) of SOM was high (>35%), whereas the CV for pH was moderate (around 15%) according to the Wilding categorized standard [32]. These findings indicated that the soil properties had wide ranges and were spatially variable within the research area to obtain high spectroscopy calibration accuracy and thus good predictive performance [33]. The similarity of summary statistics (e.g., mean, SD, and CV) from the calibration and validation sets showed that they were both able to represent the entire data set.

### 3.2. Soil Spectral Characterization

**Figure 3:**
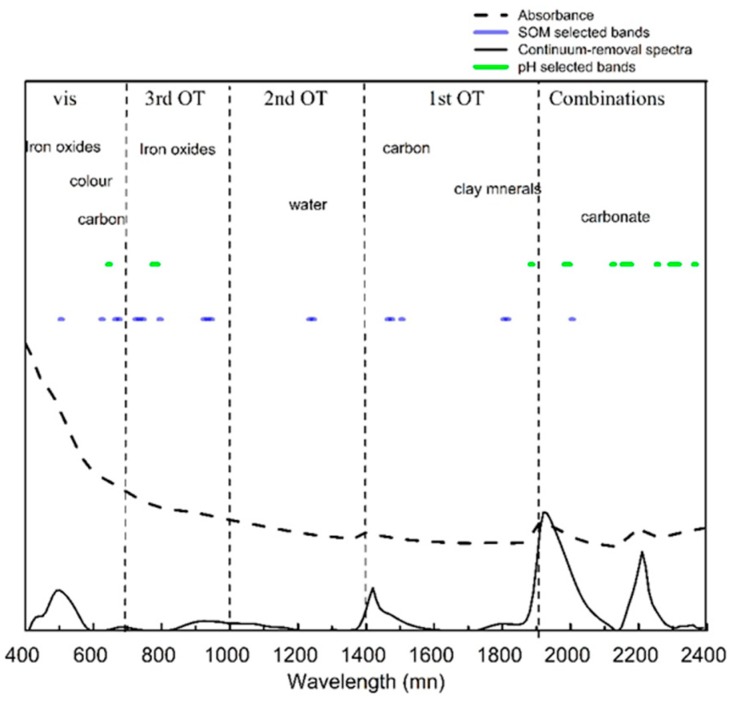
Mean absorbance spectra and their continuum-removal spectra and GA selected bands of SOM and pH in the calibration dataset.

The averaged spectra of 350 calibration samples measured in the laboratory are shown in Figure 3. Five apparent absorption regions can be seen in the absorbance spectra and their continuum-removal spectra. Absorptions near 600 and 900 nm are primarily associated with some minerals containing hematite and goethite [34], while those near 600 nm also result from chromophores and the darkness of organic matter [4].

Two prominent absorption features at about 1400 and 1900 nm were caused by the O-H functional group of the free water region [35]. The absorption features at about 1400 nm (1395 to 1415 nm) are due to overtones of the O–H stretch vibration near 2778 nm, while those near 1900 nm reflect a combination of O–H stretching and H–O–H bending in water molecules trapped in the crystal lattice [36]. The apparent absorption near 2200 nm is caused by Al–OH bending plus O–H stretch combinations [4].

**Figure 4:**
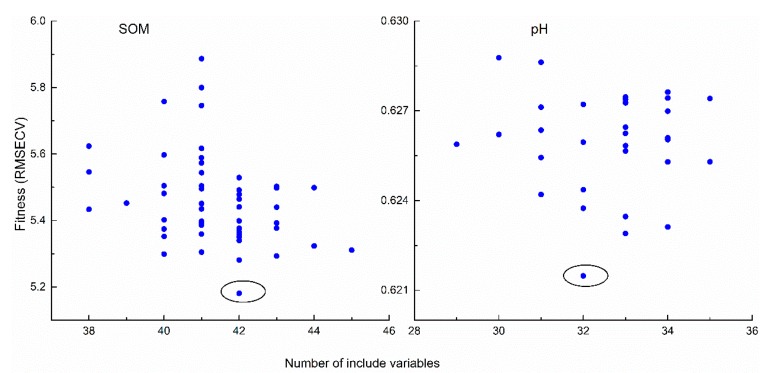
Fitness (RMSECV) versus number of variables used by each individual from genetic algorithm (GA) 10 times.

The best variables selected by GA on the basis of the RMSECV of pH and SOM used as a fitness function in GA analyses can be seen in Figure 4. The lowest values of the RMSECV of pH and SOM were 0.62 and 5.20 when 32 and 42 variables (circle) were selected, respectively. These optimal variables were used to build four models (Figure 4). These models were also used to compare the model that used the full bands to find the best machine learning methods for predicting pH and SOM.

### 3.3. Predictive Accuracy of the Machine Learning Models

**Figure 5:**
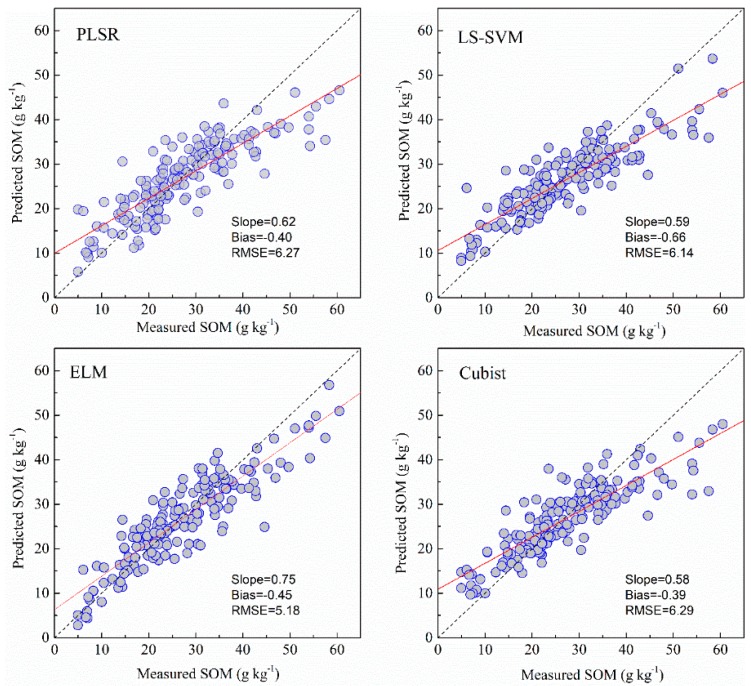
Predicted values plotted against measured values of the validation set for SOM using the PLSR, LS-SVM, ELM and Cubist methods with full bands.

**Figure 6:**
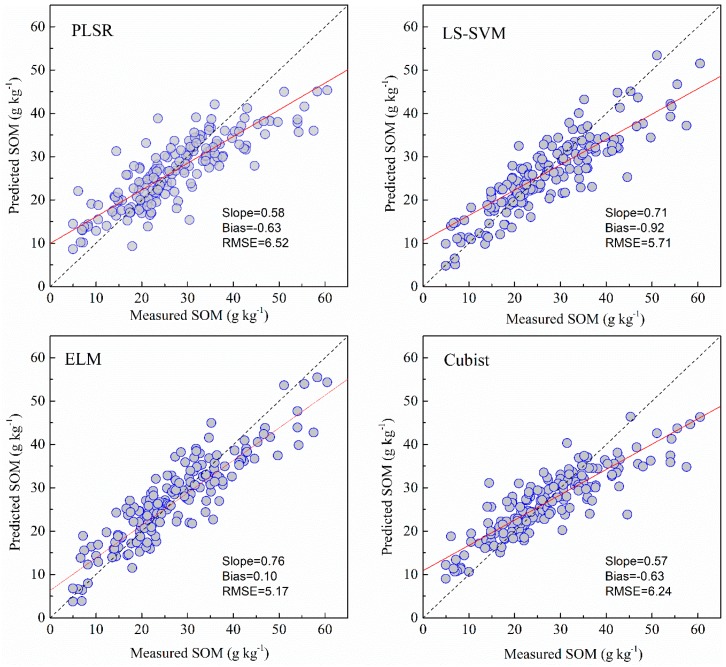
Predicted values against measured values of the validation set for SOM using the PLSR, LS-SVM, ELM and Cubist methods with bands reduced by GA.

**Figure 7:**
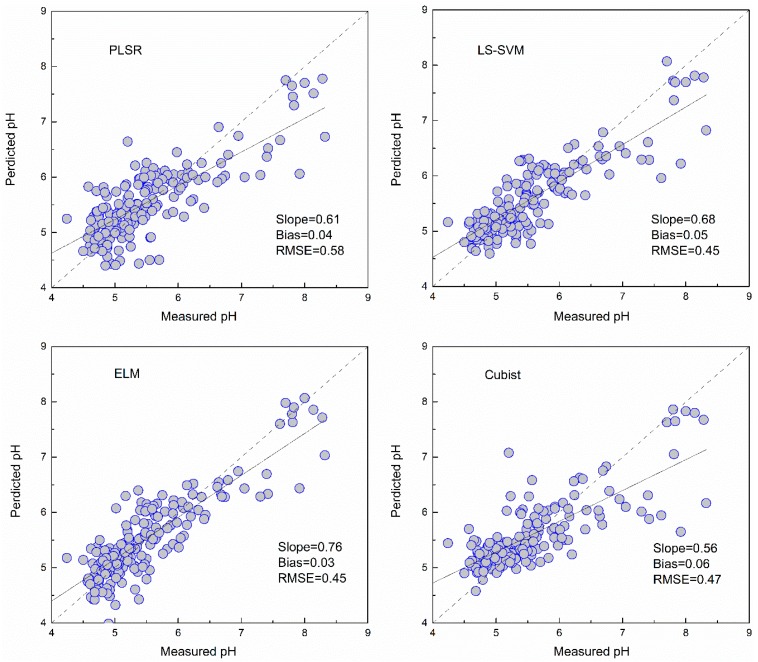
Predicted values against measured values of the validation set for pH using the PLSR, LS-SVM, ELM and Cubist methods with full bands.

**Figure 8:**
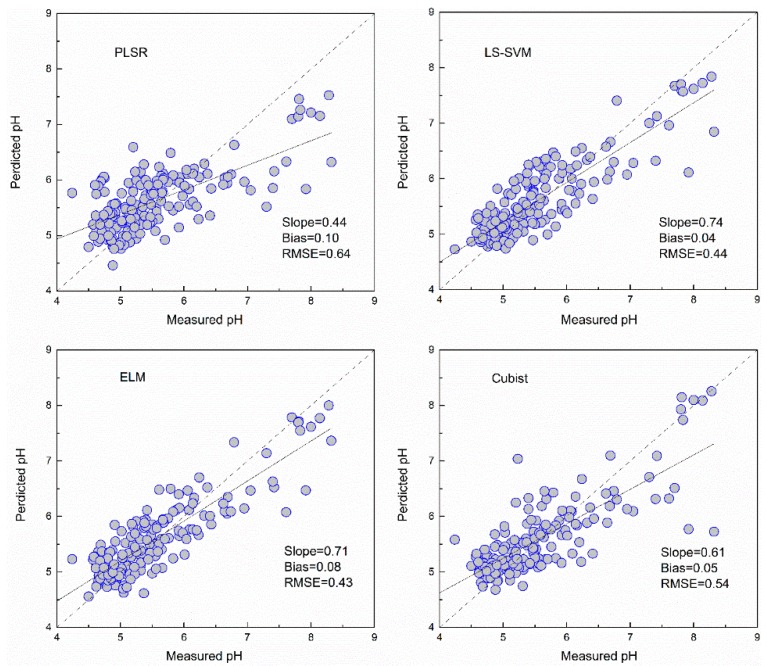
Predicted values against measured values of the validation set for pH using the PLSR, LS-SVM, ELM and Cubist methods with bands selected by GA.

Full spectral bands and bands selected by GA were used to calibrate and validate against the measured SOM and pH using PLSR, LS-SVM, ELM and Cubist models. The descriptive regression statistics of the predicted vs. measured soil properties are provided in Table 3 and the scatterplots of the predicted vs. measured soil properties for the different models are illustrated in Figure 5, Figure 6, Figure 7 and Figure 8. When using full spectral bands, the best prediction for SOM was obtained using the ELM model for both cross validation (R^2^ of 0.87 and RMSE of 3.78) and independent (R^2^ of 0.81; RMSE = 5.18; RPIQ = 2.83). With other methods using the full bands, the RMSE of the prediction in validation in ELM model was reduced by 18–21%. The PLSR and Cubist had similar prediction accuracy (RMSE = 6.27 and 6.29, RPIQ = 2.33 and 2.32).

The results of four models of SOM with the selected bands obtained by GA algorithm are listed in Table 3. The GA algorithm reduced the number of wavelengths greatly from the original 201 to 42. The prediction performance of the Cubist, ELM and LS-SVM methods based on GA selected bands was slightly improved (RMSE was reduced by 0.64%, 0.19% and 7.53% for Cubist, ELM and LS-SVM, respectively) relative to models based on full bands, while the models of PLSR based on GA selected bands were slightly reduced (RMSE was increased by 3.83%). Overall, the models that used the GA selected bands performed almost as well as those developed with full wavelengths using the same model.

The pH values predicted using the four models with the full bands and selected bands are shown in Table 3. Among these models, LS-SVM and ELM using full bands produced similar results during cross-validation and independent validation. The PLSR had the poorest result in prediction (RMSE = 0.58, RPIQ = 1.61).

The PLSR and Cubist models based on GA selected bands for pH showed poorer performance than those based on full bands, as indicated by the RMSE increasing by 9.32% and 10.01%, respectively. The LS-SVM based on GA selected bands produced similar predicted results, with a RMSE of 0.44. The ELM using GA selected bands had the best prediction, with a RMSE of 0.43 and RPIQ of 2.15.

### 3.4. Comparison of Model Performance

As shown in Figure 5, Figure 6, Figure 7 and Figure 8 and Table 3, the ELM model outperformed the PLSR, LS-SVM and Cubist models for prediction of SOM, regardless of whether the full bands (R^2^ = 0.81, RMSE = 5.18, RPIQ = 2.83) or reduced bands (R^2^ = 0.81, RMSE = 5.17, RPIQ = 2.87) were used. The LS-SVM and ELM for pH prediction using the full bands (Table 3) produced similar results (R^2^ = 0.74, RMSE = 0.45 and PRIQ = 2.07) and outperformed the PLSR and Cubist models. When the reduced bands were used, ELM had the best accuracy for prediction of pH (R^2^ = 0.76, RMSE = 0.43, RPIQ = 2.15).

## 4. Discussion

### 4.1. GA Selection

When compared with the full bands, GA selected the smallest number of effective variables (*n* = 42) as input data matrices of the four models obtained similar prediction accuracy for SOM (Table 3). These results show that the GA selection method is necessary and that a few chemically meaningful effective wavelengths can be used to obtain better predictions. Many previous studies have shown that better predictions could be obtained by GA [2,37]. Nevertheless, when compared with full bands, the applications of GA with four models did not lead to much better results (Table 3) as indicated by the poorer results obtained upon cross-validation in the calibration set. When compared using the full bands, the performance of models using reduced bands became worse in terms of lower R^2^ and larger RMSE in calibration (Table 3). These findings could be explained by the fact that GA tended to remove some useful variables during calibration when operating on the complex full-spectrum [38]. However, it is interesting to note that there were no significant differences in the validation, although some differences in the model performance of calibration were observed when the GA reduced bands were used for pH. Given the prediction results of SOM and pH, use of the GA selected bands is feasible. This is because reducing large spectral data sets to parsimonious representations can provide deeper insight into the spectral predictive mechanisms and the most important can apt to more efficient storage, computation and transmission [39].

### 4.2. Performance of Models

The four machine learning methods with two variables provided different prediction accuracies for SOM and pH. Based on the results and the R^2^, RMSE and RPIQ values in the prediction dataset, it can be concluded that the nonlinear methods performed better than the linear methods (PLSR). Non-linear relationships between the vis-NIR spectral data and soil variables have been demonstrated in many studies [40,41,42].

The non-linear ELM method showed the best performance, while the LS-SVM performed slightly better than the Cubist model. The scatterplots of the predicted vs. measured soil properties illustrated in Figure 5, Figure 6, Figure 7 and Figure 8 show that PLSR did not predict the higher and lower values of SOM and pH well when compared with the LS-SM, ELM and Cubist methods. The Cubist model and LS-SVM succeeded in predicting the high nonlinearities with the full and selected wavelengths when compared with the PLSR. The prediction values in the low and middle ranges were more compact; however, the LS-SVM method showed better prediction performance ability than the Cubist model, which is similar to the results reported by Morellos et al. [14].

In this study, one obvious feature of the ELM was that it was more accurate at predicting the higher content of SOM and pH than other models. The successful prediction performance of the ELM may have resulted from the important characteristics of this technique; namely, the arbitrarily assigned input weight and bias and the analytical determination of the output weights of the single-hidden layer feedforward neural networks [43]. This technique can prevent the ELM from being constrained to extract relevant information using non-linearly connected neurons [44]. The use of fewer user-defined parameters in the ELM allows it to have better generalization capability; therefore, the ELM can avoid changes in complex parameters such as learning rates, learning epochs, stopping criteria, and local optima [45]. Moreover, when used in a limited training set, the ELM can learn N distinct observations that use almost any nonlinear activation function approximation with most N hidden nodes.

Unlike ordinary neural networks, the ELM is a suite of machine learning techniques with hidden neurons that do not need to be tuned and there is no need to consider theories such as neural network generalization, control, matrix and linear system theory [46]. In this study, we selected the apt hidden neurons by minimizing the RMSE in the training dataset, which minimized the training errors proposed by Schmidt et al. [47]. The generalization performance of RMSE tends to become worse when too few or too many nodes are randomly generated. However, the generalization performance of the ELM is very stable over a wide range of hidden nodes (Figure 3), which is similar to the results reported by Huang et al. [43]. The ability of ELM’s potential can be used efficiently with generalized single-hidden-layer feedforward network in this study in prediction the content of SOM and pH. In the future, some ELM will also be used for multi-hidden-layer feedforward networks or implemented by various nonlinear activation and kernel functions.

## 5. Conclusions

In this study, we evaluated the ability of four machine learning techniques (PLSR, LS-SVM, ELM and Cubist) to predict the contents of SOM and the pH of paddy fields in the laboratory based on spectroscopy using the full bands and bands reduced by GA. The ELM models with the full bands and bands reduced by GA outperformed the PLSR, LS-SVM and Cubist models for SOM predictions, whereas the ELM and LS-SVM with full bands had the same prediction results for pH. The ELM outperformed other models when using bands reduced by the GA algorithm. Overall, when the same models used the full bands and reduced bands there were no significant differences in prediction, while non-linear multivariate models were superior to linear models. The best predictions were obtained by ELM with bands reduced by GA for SOM (R^2^ = 0.81, RMSE = 5.17, RPIQ = 2.87) and for pH (R^2^ = 0.76, RMSE = 0.43, RPIQ = 2.15). When compared with using the full bands (i.e., ELM for pH: R^2^ = 0.72, RMSE = 0.45, RPIQ = 2.07), a minor increase in prediction for SOM and pH was obtained using the reduced bands (i.e., ELM for pH: RMSE reduced from 0.45 to 0.43); however, the number of wavelengths was greatly reduced (i.e., ELM for pH:201 to 32). Thus, we recommend adopting the ELM using reduced bands to predict the soil properties (SOM and pH) of paddy soil in the middle-lower Yangtze Plain.

## Figures and Tables

**Table 1:** Basic properties of the soil samples in the middle-lower Yangtze plain.

Texture	Sample Number	Crop	Parent Material
Clay	83	Idle field, Silkworm	Acidic crystalline
Clay loam	220	Rice	Alluvial deposit
Loam	120	Rice	Alluvial deposit
Sandy loam	100	Grass, Idle field	Red sandstone

**Table 2:** Statistics describing organic matter content (SOM, g/kg) and pH measured in the laboratory.

Properties	Dataset	N	Range	Mean	Median	SD	CV	Skewness	Kurtosis
**SOM**	All	523	2.44–60.50	25.97	24.90	10.92	42.05%	0.44	0.14
	Calibration	350	2.44–60.50	25.43	24.67	10.56	41.53%	0.36	−0.01
	validation	173	4.96–60.50	27.07	25.5	11.57	42.74%	0.53	0.24
**pH**	All	523	3.92–8.60	5.52	5.25	0.86	15.40%	1.54	2.20
	Calibration	350	4.32–8.60	5.53	5.23	0.84	15.22%	1.64	2.50
	validation	173	3.92–8.32	5.52	5.32	0.87	15.81%	1.37	1.75

N, sample size; SD, standard deviation; CV, coefficient of variation (%).

**Table 3:** Accuracy of different methods for prediction of SOM and pH based on full bands and GA selection bands in the calibration datasets and validation datasets.

Properties	Methods	Bands	Calibration •		Validation			
			R^2^	RMSECV	R^2^	RMSE	ME	RPIQ
SOM	PLSR	full	0.79	4.81	0.72	6.27	−0.40	2.33
		GA	0.76	5.20	0.71	6.52	−0.63	2.24
	LS-SVM	full	0.83	4.43	0.76	6.14	−0.66	2.39
		GA	0.86	3.91	0.77	5.71	−0.59	2.56
	ELM	full	0.87	3.78	0.81	5.18	−0.36	2.83
		GA	0.89	3.71	0.81	5.17	−0.28	2.87
	Cubist	full	0.76	5.07	0.74	6.29	−0.39	2.32
		GA	0.78	5.08	0.76	6.25	−0.33	2.33
pH	PLSR	full	0.67	0.48	0.57	0.58	0.04	1.61
		GA	0.46	0.62	0.48	0.64	0.09	1.46
	LS-SVM	full	0.82	0.36	0.74	0.45	0.03	2.07
		GA	0.74	0.43	0.75	0.44	0.02	2.08
	ELM	full	0.85	0.33	0.74	0.45	0.03	2.07
		GA	0.72	0.45	0.76	0.43	0.01	2.15
	Cubist	full	0.70	0.49	0.72	0.47	0.03	1.98
		GA	0.64	0.57	0.62	0.54	0.05	1.71

• R^2^ and RMSECV are result of the cross validation in calibration.
